# Effect of COVID-19 Pandemic on Knowledge, Attitude and Practices Towards Antenatal Care Among Antenatal Women: A Study From a Tertiary Care Hospital in Delhi, India

**DOI:** 10.7759/cureus.52618

**Published:** 2024-01-20

**Authors:** Sheeba Marwah, Poornima Sharma, Shiwani Tripathi, Divya Arora, Ambika Agarwal, Abhigya Malik, Harsha Gaikwad, Bindu Bajaj

**Affiliations:** 1 Obstetrics and Gynaecology, Vardhman Mahavir Medical College and Safdarjung Hospital, New Delhi, IND

**Keywords:** covid-19, antenatal care, practices, knowledge, attitude, sars cov2

## Abstract

Objective: Effect of the COVID-19 pandemic on knowledge, attitude, and practices toward antenatal care among antenatal women.

Design: Prospective observational study.

Method: After taking written and informed consent, 3000 term/near-term SARS CoV2-negative antenatal women admitted to the hospital for emergency were enrolled; excluding those in advance labour or critically ill. An interview was conducted and a knowledge, attitude, and practices (KAP) questionnaire was filled out based on verbatim answers. All women were then given individualized antenatal and postnatal care as per hospital protocols and discharged accordingly. The data obtained during the study was recorded on predesigned case proforma and analysed at the end of the study using the SPSS v. 23 software, after the application of appropriate statistical tests.

Main result: All women knew about the pandemic and its signs and symptoms along with precautions to be taken. Most of the women 2652 (88.4%) thought that they were at increased risk of contracting an infection during pregnancy and 2208 (73.6%) thought that coronavirus can harm the baby and will increase the risk of pregnancy. Awareness of nearby health facilities providing antenatal care was in 71.2% and 94% were aware of functional outpatient department services but only 1.4% were aware of teleconsultation services. About 2094 women have had any ANC visits. All of them knew that taking iron, Ca and vitamin supplements and getting an ultrasound and investigations were necessary but only 1524 (50.8%) took these supplements regularly, 1752 (58.4%) got their ultrasound done and 41.6% got investigations done. Two thousand four hundred thirty-six (81.2%) women had this fear that they would contract COVID-19 infection during their visit to the hospital. All the respondents of our study wanted to have hospital delivery and knew that it was necessary to have ANC registration and none of them wanted to have home delivery.

Conclusion: Mastering correct knowledge will foster a positive attitude among antenatal women and will not only prevent disease transmission but also improve pregnancy outcomes.

## Introduction

The coronavirus disease 2019 or COVID-19 pandemic has spread all over the world, including India. Affecting all people alike, this novel coronavirus has attacked all sections of society, the young, old and also pregnant women. It spreads via respiratory droplets released by coughing or sneezing by an infected person, by contacting contaminated surfaces or aerosols generated during procedures like intubation and bronchoscopy [1]. It mainly affects the respiratory system leading to symptoms such as fever, cough, shortness of breath, alteration in taste and smell and coagulation abnormalities whilst 60 to 70% of people may be asymptomatic carriers [2,3].

People are seeking more and more information about the pandemic and forgetting about their comorbidities and otherwise routine health check-ups. The use of face masks, hand sanitiser, frequent hand washing and social distancing became the new normal with wide acceptance among all strata of the population [4]. New policies and evolving guidelines and all healthcare systems were centred on the novel coronavirus. All elective procedures, routine outpatient services and procedures were stalled. Not only this, ill-equipped hospitals, and inadequate medical and epidemiological training also posed challenges. Antenatal care (ANC) was no exception, with the routine antenatal clinics closed and no new bookings, pregnant women were unable to have even the minimum of four antenatal visits as recommended by WHO. Jeopardised ANC may also include missed vaccines and missed screening opportunities in pregnant women. Newer modalities such as telemedicine, providing virtual healthcare with the use of digital technologies came into being [5]. However, in India both awareness and availability of such facilities are meagre. If used judiciously, telemedicine can prove effective in ANC reaching out to more women without hampering safety and imparting their knowledge to identify warning signs and facilitate referral and pick-up services. Additionally poorly developed infrastructure and inadequately prepared healthcare professionals along with limited finances were also inhibiting.

The uncertainty regarding the further course of the pandemic and possible resurgence in coming months and insufficient information or misinformation spread by various media has created fear and anxiety among common people. Sudden loss of jobs, income and anxiety associated with the lockdown forced people to thrive with limited resources and even migrate to their native places, thus making their health the last priority. With a progressively rapid rise in the number of cases during the COVID-19 pandemic and the mounting fear among the general population regarding this disease, most women avoid routine antenatal visits and investigations whenever feasible [6]. This leads to non-recognition of high-risk conditions which are common in obstetrics and potential upshots. The initial lockdown and the movement restrictions associated with it, along with almost all healthcare facilities either changing into COVID-dedicated centres or establishing simultaneous COVID care areas, left people in a difficult situation further discouraging outpatient visits. This further compromised ANC in the underprivileged section of society. This study will help us to formulate policies tailored to our population for the provision of the best possible care to women catered by the public sector hospitals. These population strata mostly belong to lower socioeconomic status and have no access to teleconsultation services. By further delving into the awareness levels, behaviour and perceptions of pregnant individuals concerning the virus, we would be able to glean critical information for both healthcare providers and policymakers. Thus, we aim to study the effect of the COVID-19 pandemic on maternity services in antenatal women through their knowledge, attitude and practice toward routine healthcare in order to streamline and ensure the provision of respectful maternal care.

## Materials and methods

Study cohort

In this study, 3000 antenatal patients admitted to the obstetric wards were recruited over a period of six months. It is a prospective observational study done at the Department of Obstetrics & Gynaecology Vardhman Mahavir Medical College and Safdarjung Hospital, New Delhi, by interviewing these near-term antenatal women and getting filled out KAP questionnaire based on verbatim answers by them.

Inclusion criteria

Antenatal women of all age groups and demographic areas were included at or near-term visiting hospitals for ANC.

Exclusion criteria

Women who were critically ill, in advanced labour or who were SARS CoV-2-positive anytime during hospital stay and refused to give consent, were excluded from the study.

Study protocol

After obtaining ethical clearance from the institutional ethics committee, written informed consent was taken from all antenatal women fulfilling the eligibility criteria. An interview was conducted and a KAP questionnaire was filled. Any difficulty in understanding any question or any query was resolved there itself. A detailed history and clinical examination were carried out on each of them and they were managed as per hospital protocol. Each patient received routine antenatal and postnatal care and was discharged as per hospital protocols.

Data recording and statistical analysis

The data obtained during the study was recorded in a predesigned case proforma and entered into an Excel spreadsheet. It was analysed at the end of the study using the SPSS v23 version of the software, after the application of appropriate statistical tests. Descriptive statistics were elaborated in the form of means/standard deviations and medians/IQRs for continuous variables, and frequencies and percentages for categorical variables. Data was presented in a graphical manner wherever appropriate.

## Results

Sociodemographic details

Data from a total of 3000 COVID-negative antenatal women was analysed with a 95.2% response rate among 3150 recruited women. The present study revealed that 58.6% of women belonged to the 26-35 years of age group, with the mean age being 26.69 ± 3.66 years. Most of the women in the study population were illiterate (66.8%). More than 80% were residing in rural areas. All of them were above 34 weeks of period of gestation (POG) and more than 75% of participants were at 37-40 weeks of POG. Around 65% of them were multigravida and four-fifths of them were housewives (Table 1).

**Table 1:** Sociodemographic details of participants

Basic Details	Mean ± SD, Frequency (%)
Age (Years)	26.69 ± 3.66
Age Group	
18-25 Years	1206 (40.2%)
26-35 Years	1758 (58.6%)
More than 35 Years	36(1.2%)
Qualification	
Illiterate	2004 (66.8%)
High School	834 (27.8%)
Intermediate	150 (5.0%)
Graduate	12 (0.4%)
Residence	
Urban	2592 (86.4%)
Rural	408 (13.6%)
Religion	
Hindu	2664 (88.8%)
Muslim	318 (10.6%)
Sikh	18 (0.6%)
Mean period of gestation (POG) (In Weeks)	38.50 ± 1.92 || 38.86 (38.00-39.57) || 28.57 - 41.29
POG	POG
Less than 34 Weeks	120 (4.0%)
34-37 Weeks	192 (6.4%)
37-40 Weeks	2298 (76.6%)
≥40 Weeks	≥40 Weeks
Gravidity	
Multigravida	1968 (65.6%)
Primigravida	1031 (34.4%)
Occupation	
Housewife	2406 (80.2%)
Working	594 (19.8%)

Knowledge of COVID-19 of our study population

More than 90% of them were aware of the disease through news, friends and family members. All were well versed with the clinical spectrum of COVID-19 like fever, cough and breathlessness; but they were lacking in knowledge about atypical presentation of the infection. Maximum women were learned in the preventive measures to avert COVID-19 infection. Only 18.4% had knowledge that an asymptomatic person can also transmit this infection. Three-fourths of the study populace were mindful of home remedies that were used for the prevention or treatment of coronavirus infection, however, only a few had knowledge of medicines (0.2%) and possible deterioration during the course of infection may require ICU admission (21.4%).

Around 88.4% of women were apprehensive about the increased risk of contracting an infection during pregnancy and 73.6% thought that it could harm their baby. About 81% of women knew the importance of ANC visits and the need for ultrasound examination and immunisation. About 71.2% were aware of nearby health facilities providing ANC to non-infected women during the pandemic and 94% of women knew about functional outpatient department (OPD) services. Only 1.4% were aware of the teleconsultation services (Table 2).

**Table 2:** Results of the knowledge-related questionnaire

	Questions	Yes	No	Don’t know
K1	Are you aware of the coronavirus pandemic?	3000 (100.0%)	0 (0.0%)	0 (0.0%)
	How are you getting information and updates about coronavirus?			
K2	News	3000 (100.0%)	0 (0.0%)	0 (0.0%)
K3	Friends and family members	2796 (93.2%)	204(6.8%)	0 (0.0%)
K4	Healthcare workers	1788 (59.6%)	1212(40.4%)	0 (0.0%)
K5	WhatsApp	612 (20.4%)	2388 (79.6%)	0 (0.0%)
K6	Aarogya Setu app	684 (22.8%)	2316 (77.2%)	0 (0.0%)
	What are the symptoms of coronavirus infection?			
K7	Fever	3000 (100%)	0 (0.0%)	0 (0.0%)
K8	Cough	3000 (100%)	0 (0.0%)	0 (0.0%)
K9	Breathlessness	3000 (100%)	0 (0.0%)	0 (0.0%)
K10	Altered taste/smell	858 (28.6%)	2142 (71.4%)	0 (0.0%)
K11	Pink eye	0 (0.0%)	3000 (100%)	0 (0.0%)
K12	Loose stool	0 (0.0%)	3000 (100%)	0 (0.0%)
K13	Runny nose	2388 (79.6%)	612 (20.4%)	0 (0.0%)
	How can you prevent coronavirus infection?			
K14	Staying at home	3000 (100%)	0 (0.0%)	0 (0.0%)
K15	Washing hands regularly with soap and water	3000 (100%)	0 (0.0%)	0 (0.0%)
K16	Using hand sanitiser	3000 (100%)	0 (0.0%)	0 (0.0%)
K17	Not touching face, nose, eyes	2292 (76.4%)	708 (23.6%)	0 (0.0%)
K18	Wearing mask	3000 (100%)	0 (0.0%)	0 (0.0%)
K19	Attending party/gathering	0 (0.0%)	3000 (100%)	0 (0.0%)
K20	Frequent hospital visits	336 (11.2%)	2658 (88.6%)	6 (0.2%)
	How is coronavirus infection treated?			
K21	Homemade immunity boosters(like turmeric milk, cinnamon, nuts)	2130 (71%)	498(16.6%)	372 (12.4%)
K22	Medicine	6 (0.2%)	2994 (99.8%)	0 (0.0%)
K23	ICU care	642 (21.4%)	2358 (78.6%)	0 (0.0%)
K24	Home isolation	2466 (82.2%)	432 (14.4%)	102 (3.4%)
K25	Supportive care	2112 (70.4%)	768 (25.6%)	120 (4.0%)
K26	Are you more at risk of contracting coronavirus infection during pregnancy?	2652 (88.4%)	318 (10.6%)	30 (1.0%)
K27	Can coronavirus infection harm your baby or increase the risk of pregnancy?	2208 (73.6%)	744 (24.8%)	48 (1.6%)
K28	Can a person who is not sick transmit this infection to you?	552 (18.4%)	2274 (75.8%)	174 (5.8%)
K29	Do you think it is necessary to get your antenatal investigations during this pandemic?	2436 (81.2%)	564 (18.8%)	0 (0.0%)
K30	Do you think ultrasounds and TT injections are needed in pregnancy?	2424 (80.8%)	570 (19%)	6 (0.2%)
K31	Are you aware of nearby health facilities providing antenatal care in pandemic times?	2136 (71.2%)	864 (28.8%)	0 (0.0%)
K32	Are you aware of functional OPD services around your home?	2820 (94%)	180 (6.0%)	0 (0.0%)
K33	Are you aware and have access to teleconsultation services?	42 (1.4%)	2958 (98.6%)	0 (0.0%)

Attitude towards COVID-19 in study population

All women knew the importance of blood tests and ultrasound examination, in addition to iron, folic acid and calcium supplementation. Though all of them thought about registering themselves in antenatal clinics during pregnancy, only 67.0% knew the significance of regular ANC visits. Around 80% of them were afraid of catching an infection during hospital visits. All of them agreed with the idea that social gatherings can be avoided during pregnancy. None of them assented to the concept of home delivery (Table 3).

**Table 3:** Results of the attitude-related questionnaire

	Questions	Yes	No	Don’t know
A1	Did you plan your current pregnancy?	1752 (58.4%)	1248 (41.6%)	0(0.0%)
A2	Do you think regular antenatal visits are needed during pandemic time?	2010 (67.0%)	990 (33.0%)	0 (0.0%)
A3	Do you think taking iron, calcium and vitamin supplements is necessary during pregnancy?	3000 (100.0%)	0 (0.0%)	0 (0.0%)
A4	Do you feel ultrasound and blood tests should be done in pandemic time?	3000 (100.0%)	0 (0.0%)	0 (0.0%)
A5	Are you afraid that you might get a coronavirus infection during your visit to the hospital?	2436 (81.2%)	564 (18.8%)	0 (0.0%)
A6	Do you think that this pandemic will end by the time you deliver?	1362 (45.4%)	1104 (36.8%)	534 (17.8%)
A7	Do you think it is necessary to get yourself registered in antenatal clinic during pregnancy?	3000 (100.0%)	0 (0.0%)	0 (0.0%)
A8	Do you feel social gatherings should be attended during pandemic times?	0 (0.0%)	3000(100.0%)	0 (0.0%)
A9	Would you prefer home delivery during pandemic times?	0 (0.0%)	3000 (100.0%)	0 (0.0%)

Practices of study population during COVID-19

Among the 3000 enrolled women in our study, 30.2% had no prior ANC visits during pregnancy. Though all of them had taken precautionary measures while going outside only 54.4% maintained social distancing during hospital visits. Approximately 50% of women took iron, vitamin supplements, got ultrasound examinations and blood investigations done during the pandemic. Around 70% of them deferred their ANC visits while 67.4% postponed the ultrasound examination. All of them planned for institutional delivery (Table 4).

**Table 4:** Result of practice-related questionnaire

	Questions	Yes	No
P1	Have you had any antenatal visits in this pregnancy?	2094 (69.8%)	906 (30.2%)
P2	Are you maintaining a metre distance while in the OPD queue?	1632 (54.4%)	1368 (45.6%)
P3	Are you using face mask and other precautions when you go outside?	3000 (100.0%)	0 (0.0%)
P4	Have you had any contact with a coronavirus-positive patient accidentally?	150 (5.0%)	2850(95.0%)
P5	Have you taken your iron and vitamin tablets regularly?	1524 (50.8%)	1476 (49.2%)
P6	Do you get ultrasound and blood investigations done during the antenatal period?	1752 (58.4%)	1248 (41.6%)
P7	Would you prefer getting delivered at hospital?	3000(100.0%)	0 (0.0%)
P8	Did you defer your antenatal visit?	2100(70.0%)	900 (30.0%)
P9	Did you defer your ultrasound?	2022 (67.4%)	978 (32.6%)

## Discussion

This COVID-19 pandemic had a great impact on all sectors of society and population. The health sector was worst hit, all the resources were regularly being channelled towards the pandemic, thereby marginalizing other healthcare needs of the population including antenatal services in the country. Owing to the lockdown and restriction in transport services, amidst a backdrop of uncertainty looming large around the novel coronavirus, routine ANC was further compromised [7]. As per WHO, antenatal women should have a minimum of eight visits, which during the COVID pandemic times were reduced to six in-person and two virtual contacts, that too when absolutely necessary [8]. By exploring their knowledge, practices, and attitudes regarding COVID-19 during pregnancy, this study has yielded valuable insights that can significantly impact maternal and foetal health.

Nearly all pregnant women had a commendable level of knowledge regarding the pandemic, with mass media emerging as an imperative tool for information dissemination. These results were parallel to a cross-sectional study conducted in Ethiopia, where all participants were aware of COVID-19, but only half possessed a satisfactory level of knowledge about the disease [9]. Another cross-sectional study from the same country found that only 54.84% of participants had adequate knowledge of the disease, surpassing the percentage observed in a similar Egyptian study(16.39%) [10,11]. The variations in these results could be attributed to differences in the study settings, literacy and occupation. The present study was exclusively conducted in an urban environment with more than 80% of women hailing from these areas, equipped with mass media and widespread social media exposure, similar to the Egyptian research observations. Based on these findings, the development of targeted educational interventions to enhance public awareness is recommended, besides ensuring how well and rapidly this accurate information would be circulated to this vulnerable population without creating unnecessary panic.

A vast majority of women demonstrated awareness of preventive measures and the significance of social distancing, emphasizing a heightened understanding of the necessity for precautionary measures to curb the spread of the virus. This finding resonates with the global emphasis on public health measures during the pandemic (World Health Organization, 2020) [12]. However, only a minority knew how drugs related to the infection were used. This was in congruence with a recent meta-analysis [13]. This emphasizes the challenges in disseminating detailed medical information to the masses as there are chances of its misuse and misinterpretation by a few antisocial elements by creating unwanted fear among the less learned sections of the community. A considerable majority expressing concerns about the risk of acquisition of infection during pregnancy and its potential harm to their unborn child underscores the importance of informational and support initiatives aimed at addressing and alleviating anxiety among pregnant individuals.

Fortunately, all women were cognizant of the significance of ANC visits, underlining the recognition of regular check-ups for maternal and foetal well-being, and the importance of ultrasound examinations and immunization, reflecting a strong understanding of essential healthcare practices during pregnancy. Also, a substantial number were aware of nearby functional health facilities and the availability of OPD services, but they were lacking in teleconsultation services. In their findings, an interrupted time-series analysis conducted in Victoria (Australia) reported telehealth-integrated ANC leading to the reduction of in-person consultations by 50% without compromising pregnancy outcomes [14]. Another study reported 25% usage of telehealth services among participants [15]. This can be inferred as a reluctance to adapt to a new lifestyle that their concerns and fear may remain imperceptible during telecare. This mirrors the conclusion drawn by investigators hailing from China, with an increased overall pooled prevalence of anxiety during the pandemic that may be attributed to the same [16]. Addressing and mitigating these fears should be a priority to ensure that pregnant women feel safe and confident in seeking the necessary medical care during any disaster in the future. Thus, the policymakers must lay down the guidelines pertaining to teleconsultation at all times; which could be particularly relevant during the time of any disaster or situation leading to the restriction of the vulnerable population addressing to the stress debriefing and management. This would be particularly of assistance in remote areas with difficult access to healthcare facilities and transportation.

Attitudes toward COVID-19 disease in the present study during pregnancy further provide a deeper insight into the emotional and psychological aspects involved, especially the impact on mental health. Addressing these attitudes is crucial for providing adequate support, counselling and mental health resources to ensure the well-being of the pregnant population. The participants were well-versed in the significance of diagnostic procedures for feto-maternal monitoring, the necessity of iron and calcium supplements; thus reflecting a comprehensive understanding of the essential obstetric care required by them. This sentiment echoed the findings of a systematic review from South America [17]

Another remarkable finding is that the awareness was put very well into practice by women in the study, which was similar to a cross-sectional study conducted in the Philippines, establishing prior pregnancy experiences as the positive predictors of adequate ANC [18]. This suggests a general adherence to the importance of seeking prenatal care, despite the challenges posed by any restrictive future health condition. An affirmation of the practice of social distancing by the women resonates with the aforementioned systematic review. This finding resonates with the systematic review of maternal and perinatal outcomes of COVID-19-infected pregnant women, which noted an increased awareness of social distancing measures among pregnant women seeking healthcare services [17]. Unfortunately, a potential gap in the continuity of comprehensive ANC was observed with only half of the women practising intake of supplements, which might be due to various factors like low literacy skills, restricted access to mass media due to limited internet availability in remote areas, concerns about infection risk, or disruptions in healthcare services and transportations during lockdown. Formulation of strategies to improve understanding of disaster risks, hazards and vulnerability is endorsed furthermore, enhancing the disaster preparedness for effective response in coming times without any public panic and social disruption.

Interestingly, all participants expressed a preference for hospital delivery. This aligns with global trends [19]. The strong preference for institutional deliveries among the study populace emphasized the perceived safety and assurance associated with professional medical assistance during childbirth. Though a majority of participants knew the importance of antenatal visits and investigations, still most of the study population postponed their ANC visits and ultrasound appointments. This raises important questions about the perception of information received by women. It can be related to the myths of the spread of infection during hospital visits as well as the difficulty of accepting new norms. Lockdown and difficult access to transport medium can also be contributory factors to this. Identifying any gaps or areas of improvement in antenatal practices is advocated, which can help in forming public health strategies to promote optimal health outcomes for both the pregnant individual and the unborn child. This will help them to respond timely and appropriately in cases of emergency.

Overall, the results of the aforementioned accentuate the complex interplay of factors influencing healthcare behaviours during any adverse situation like the COVID-19 pandemic. It is praiseworthy to see the commitment towards ANC visits and precautionary measures, the observed variations in the uptake of supplements and diagnostic procedures warrant further research. This infers that having adequate knowledge doesn’t always lead to the adoption of new practices. These observations provide an opportunity for healthcare professionals to design regulatory strategies, mass awareness campaigns, the role of social media, public health interventions and healthcare policies to improve the knowledge among target populations aimed at optimizing maternal and foetal health in disaster management in the future.

Strength and limitation

The present study comprehensively examines various facets of the COVID-19 pandemic and its impact on ANC. Additionally, the research was conducted at the peak of the disease, providing valuable insights into knowledge and practice among pregnant women. Taking a leaf out from this study, health education campaigns can be designed to tackle such situations in future. The primary drawback of the current study lies in its restriction to a single institution and a relatively small cohort size, it does not reflect the general population, which may introduce selection bias. We acknowledge the shortcomings of the present study, it is essential to recognize its strength.

## Conclusions

The findings of this study showed that most of the participants had a reasonable understanding of COVID-19 infection overall and the right mindset regarding preventive measures. However, there was a reduction in utilization of antenatal services. Thus, it’s concluded that the gaps that keep pregnant women away from receiving pertinent information need to be addressed and the necessity to improve the availability of psychological support during these delicate times. Given the risk of future health emergencies, information, education and communication (IEC) materials should be produced by policymakers and healthcare professionals along with the strategies to raise awareness among pregnant women, especially in resource-limited settings. Legal implementation of preventive practices and health education to pregnant women can be of help with improvement in healthcare delivery, allowing them to be better prepared and more resilient in coming times.
